# Topics of the Time

**Published:** 1897-11

**Authors:** 


### TOPICS OF THE TIME.

A suit to recover $40,000 damages for alleged libel, brought by Joseph A. MacKeown, an optician of No. 24 East Forty-second street, New York, against Dr. Frank Van Fleet, who, in an address before the Medical Society of the County of New York, had spoken of him by implication as a quack, was tried recently in the United States Circuit Court before Judge Wallace and a jury.

Dr. Van Fleet is chairman of the committee on legislation of the Medical Society of the County of New York, and in the course of his official duty as such comes in contact with, and naturally incites the displeasure of, each and every person who is attempting to relieve human ills by irregular and illegal measures.

The remarks complained of were made in the course of a debate over the admission to the columns of *The Medical Record* of an advertisement of MacKeown's and was reported in the newspapers. In it Dr. Van Fleet said : "I have been told the case of a man, a druggist, who died of Bright's disease. He told me he had seen this advertisement in our journal and, thinking it had our indorsement, had his eyes treated for this trouble. I hold that the advertisement was the means of hastening his death at least. A man who says he can do all things this man says he can, can do more than any physician, and he is a quack. The medical journals are overrun with these meretricious advertisements, I am sorry to say, but I want our own to be free from them. It seems to me that when a person not a physician advertises to cure headache, nervousness, sore eyes, neuralgia and other things by putting on glasses, then he violates the law as a quack. Such advertisements should be withdrawn from medical journals. If there is no legal right to withdraw them, then there is a moral right. The whole country is teeming with such quackery and, what is worst of all, when such advertisements appear in a medical journal, and especially in our own publication, it appears to have our indorsement."

Dr. Van Fleet, at the trial, testified in his own behalf that he did not know that reporters were present when he addressed the county medical society. He said that he spoke of MacKeown as he did for the reason that the latter advertised to do what he had no right to do, not being a licensed practitioner. Judge Wallace directed a verdict for the defendant.

Dr. Van Fleet is an able and fearless champion of the right and deserves to be continued on the legislative committee as long as he will consent to serve. The JOURNAL congratulates him upon winning the suit above referred to.

---

COMMENTING upon the death of a little child, treated by the so-called christian-science method, the *Peoria Medical Journal* says:

The recent occurrence in this city of the death of little Hazel Campbell has aroused a storm of condemnation and protest in the minds of the general, as well as the professional, public. In this is presented the instance of a bright and lovely child, allowed not only to die, but to suffer the agony of acute intestinal inflammation for want of that attention which medical science at the present day can readily furnish. It is well, perhaps, that such shocks be occasionally administered to the public consciousness, in order to arouse it to the sense of danger to which it is constantly exposed, but it is sad to contemplate the inhumanity and desolation of a home inflicted by such a blind and unreasoning adherence to a faith that is at best only a half truth.

The question in one of our city papers in this connection may well be asked: By what right are these people allowed to take a serious case like that and treat it? Physicians are required to pass an examination and hold a state certificate before they are allowed to practise their profession, yet these christian-scientists take a case, no matter how serious it may be, and are not touched by the law, even if the patient dies, as is the case every time, when there is anything ailing the structure of the body—anything worse than a mere functional nervous derangement, which can in a measure be removed or controlled by the hypnotic treatment given by the christian-scientists.

The question as to the duty of the state board of health in this connection is an important and serious one. The idea of exacting from one class of persons a qualification that is not exacted from another class for the self-same liberty is repugnant to all sense of justice and right, aside from the more important consideration of the welfare of the public. And yet that is exactly the status of matters in this state at the present time relative to the class of so-called healers, called "christian-scientists" by suffrage and the members of the general profession of medicine. That it is an outrage, pure and simple, on all concerned, is patent to anyone willing to give the matter even a moment's thought. That the state board of health will ever take any action in the premises is not at all likely.

In the case forming the text of the foregoing outburst, the coroner was called to take action, but refused finally to do so, with the statement that while he realised a great wrong had been done it was not his province to take any action, in that such move had been frustrated by calling in physicians just before death, the cause of which was certified to by them.

---

In scarlet fever patients it is a very important matter to determine how long after convalescence isolation should be maintained. James T. Neech, L. R. C. P., Edin., read a paper relating to this subject at the Montreal meeting of the British Medical Association, published in the *British Medical Journal*, September 25, 1897. The *Medical Review*, October 23, 1897, makes editorial comment upon the same and the importance of the subject is such as to justify us in making extended extracts from the same. Says the *Review*:

With a view of ascertaining the duration of infectiousness in convalescents from scarlet fever, Dr. Leech has gathered carefully prepared statistics in connection with cases while in hospital and the relation which their discharge from hospital bears to the occurrence of fresh cases in the district where they live. While the author admits that until the specific microbe of the disease has been isolated, its life history studied and the extreme limit of its retention in the human body experimentally proved, it will be impossible to pronounce with certainty how long after convalescence a given patient can be considered free from infection and incapable of conveying the disease. Until then physicians must be guided in dismissing patients from hospitals by the results of close observation, accurate records and carefully prepared statistics.

In this investigation the author has secured pretty accurate records of 15,000 patients and found that where the average period of isolation was forty-nine days and under the return cases averaged 1.86 per cent.; where the average period varied between fifty and fifty-six days, the average percentage of return cases was 1.12 and where isolation extended to between fifty-seven and sixty-five days the average percentage was 1.0. Although the author would not like to lay too much stress upon these figures, yet they seem to point to the conclusion that the average period of retention in hospital bears a relation to the number of return cases of the disease. The author does not ignore the possibility that an individual might become infected from some unknown source and in each case this might be coincident with the return from hospital. The stowing away of infected articles belonging to a patient has been shown to be a cause of return cases, but with improved apparatus and more perfect and thorough methods of disinfection, this probably does not influence the number of return cases so much as formerly. Making due allowance, then, for a certain number of return cases from other sources, there is little doubt in the author's mind that discharged patients in a certain percentage of cases are the direct vehicles of infection.

The author's statistical research would seem to refute the idea that, as several eminent authorities believe, patients breathing the actively infected air of a hospital during convalescence store up infective material in the cavities of nose and lungs, and by that means convey the poison outside the institution. This may possibly occur, says the author, but were it the chief cause, would not the number of return cases be even greater than it is? Moreover, in that case the percentage would not be affected by prolonged hospital isolation.

The author calls attention to the fact that the infectious material circulating in the blood must be present, in all probability, in all the secretions of the body, also the feces and urine, and the latter should therefore be disinfected. The desquamating cuticle in scarlet fever owes its infectiousness to the excretion of the glands of the skin. In scarlet fever not only the glands of the neck but also the lymphatic glands in the axilla, groin and other parts become enlarged during the earlier stages of the disease. The author inclines to the opinion that the specific poison accumulates in these glands. Later the virus passes again into the blood-stream and is eliminated during convalescence by the emunctory organs.

That a purulent discharge from the nose, ear or running sore, the usual sequelæ and complications which necessitate a prolonged period of isolation, is infectious, has been proved beyond a doubt. The mere existence, however, of such discharges is not in itself capable of prolonging the infective period. It has not yet been shown that the cessation of the period of infectiousness and the healing of a discharging surface are coincident and the author does not think that such is always the case.

The completion of desquamation is not a safe and reliable guide as to when a patient ceases to be infective, even in cases where no complication arises.

In all acute infectious diseases, where the fluids of the body contain more or less of the specific virus, a certain time must elapse before that virus can be completely eliminated.

It appears that in scarlet fever conditions exist, at least in some cases, that considerably prolong the period of complete elimination. In this respect scarlet fever seems to differ from many of the other fevers ; the idea has, therefore, suggested itself to the author that a storing up of the infectious material takes place somewhere in the body and this, perhaps, in the condition of spores.

The author recommends the establishment of convalescent wards in all fever hospitals and, with regard to the time of detention in scarlet fever, he thinks that a minimum of eight and a maximum of thirteen weeks will be sufficient until the contrary be shown by accurate methods based upon careful observation, or after the infectious agent has been proven to be a microörganism, by the study of the life history of the germ ; in other words, by bacteriological methods.

---

By a printer's error the annual death-rate for the month of September, 1897, for the City of Buffalo, in the monthly bulletin of the deparment of health, was made to read 16.11 instead of 11.61.

---

Mr. Hall Caine writes interesting stories, but occasionally he gets his therapeutics somewhat mixed, as instanced by the following, taken from the *Bristol Medico-Chirurgical Journal* for September :

In Mr. Hall Caine's last novel, The Christian, some comical perversions of medical knowledge occur. For example, the weak-minded but beautiful Polly Love is killed off by a dose of "half a grain of liquor strychnine." This is, perhaps, not quite in accordance with modern pharmacology, but Mr. Caine unfortunately omits to mention anything about the chemist who weighed out the half grain of the liquid poison. In a medico-legal matter, again, Mr. Caine has established a record, and it is evident that we are quite wrong as to the definition of a still-born child, if this record is to be maintained. In The Christian the author describes a still-born child as "one that has breathed but never cried."—*Medical Press and Circular.*

